# Emotion regulation strategies of experienced oncology nurses: a qualitative study

**DOI:** 10.5116/ijme.6921.a243

**Published:** 2025-11-26

**Authors:** Yuji Iwama

**Affiliations:** 1 Department of Nursing, National Defense Medical College, Tokorozawa, Japan

**Keywords:** Oncology nursing, emotional regulation, metacognition, qualitative study, SCAT

## Abstract

**Objectives:**

This study aimed to elucidate, through qualitative analysis, the cognitive processes by which experienced oncology nurses regulate their emotions when facing anxiety and emotional conflict in communication with patients.

**Methods:**

We employed a qualitative exploratory design using semi-structured individual interviews with six certified oncology nurses in Japan. Participants were recruited via snowball sampling and provided written informed consent. Interviews were conducted in Japanese using a pre-tested interview guide; audio data were transcribed verbatim. Data were analyzed with the Steps for Coding and Theorization (SCAT) method. Metacognitive theory—distinguishing metacognitive knowledge and metacognitive regulation—guided interpretation of the findings.

**Results:**

Analysis of 38 theoretical descriptions yielded two overarching themes: (1) anxiety and conflict in communication with cancer patients, and (2) metacognitive emotion-regulation strategies. The latter comprised two subthemes: (a) patient-oriented cognitive strategies (e.g., linguistic adjustments, trust-building, facilitating patients’ self-regulation), and (b) self-oriented cognitive restructuring (e.g., reframing dilemmas, monitoring and modulating one’s own emotional responses). These processes reflected deliberate monitoring and regulation of thinking and feelings to sustain constructive engagement with patients.

**Conclusions:**

Experienced oncology nurses use metacognition to recognize, interpret, and flexibly regulate emotions in challenging interpersonal situations. Educational implications include integrating structured metacognitive reflection alongside empathy and mindfulness training to cultivate durable, transferable coping skills. Future studies should examine this approach in diverse clinical contexts, include larger and cross-cultural samples, and evaluate longer-term outcomes in nurses’ emotional resilience and clinical practice.

## Introduction

For nurses engaged in cancer care, communication with patients is one of the most routine tasks, yet it also constitutes a significant source of difficulty.^1^^-^^8^These emotional challenges, when accumulated over time, are reported to contribute to burnout and even attrition among nurses.^9^ The root of these difficulties often lies in patients’ emotional fluctuations caused by the psychological burden of cancer.^1^^,^^4^^,^^8^ These fluctuations may provoke unexpected reactions to nurses’ involvement or lead patients to pose questions with no definitive answers, both of which can be distressing for nurses.^2^^,^^3^

To address these challenges, recent years have seen the promotion of training programs aimed at enhancing empathic capacity.^10^^,^^11^ These programs improve nurses’ immediate responses and situational coping skills, but they do not adequately foster the sustained cognitive reorganization required for professional resilience.^12^^-^^14^Gross’s process model of emotion regulation provides a useful framework for understanding this limitation.^15^ The model distinguishes between antecedent-focused regulation, applied before emotions are fully generated, and response-focused regulation, applied after emotions have arisen. Conventional nursing education has largely emphasized response-focused strategies, such as mindfulness or empathic communication to modulate reactions once they occur.^11^^,^^16^ While these approaches are valuable for stress reduction and burnout prevention, they fall short when the aim is to transform anxiety into a learning opportunity. In this regard, antecedent-focused strategies that prepare nurses to analyze and reorganize their thoughts before avoidance sets in become crucial.

Building on this theoretical distinction, metacognition provides a broader perspective on how nurses regulate their emotions. Defined as the awareness and regulation of one’s own cognitive and emotional processes, metacognition comprises both knowledge (recognition of tendencies in thinking and emotions) and regulation (monitoring and adjusting these processes).^17^^,^^18^ Applied to emotion regulation, this perspective clarifies how nurses deliberately reinterpret anxiety and restructure their responses, thereby sustaining constructive engagement with patients.

Based on the distinction between antecedent- and response focused strategies, the survey by Suzuki and Furuse offers a complementary view.^19^ They found that nurses who responded constructively in difficult situations attended closely to both their own emotions and those of their patients. This attentiveness can be interpreted as a form of preparatory thinking that enables nurses to contextualize and reorganize their responses—consistent with the antecedent-focused perspective. Such findings highlight the importance of moving beyond surface-level techniques to examine the underlying cognitive processes through which nurses manage emotionally challenging encounters.

Although strategies such as empathy, mindfulness, and attentiveness to emotions can support coping.^11^^,^^16^^,^^20^ The mechanisms through which experienced oncology nurses recognize, interpret, and regulate their own anxiety during patient encounters remain poorly understood. Even experienced oncology nurses, despite their advanced expertise and extensive exposure to clinical practice, continue to encounter such difficulties.^2^^,^^3^^,^^6^ Importantly, not all experienced nurses cope equally well: some manage to engage constructively, while others continue to struggle.^2^ We considered that these differences may arise from variations in how nurses think when confronted with anxiety. Focusing on experienced oncology nurses therefore provides an opportunity to clarify the cognitive and metacognitive processes that distinguish more effective from less effective regulation.

Clarifying how oncology nurses confront and overcome the anxiety and conflict they encounter in relationships with patients holds both practical and educational significance. Especially in daily care situations, where nurses are susceptible to emotional influence, the ability to appropriately regulate emotions and reconstruct thoughts and actions becomes a vital competency that enables more meaningful nursing practice. Because these psychological and cognitive processes are difficult to observe directly, exploring them requires methodologies that capture nurses’ conscious articulation and reflection on their experiences. A qualitative exploratory approach is therefore particularly suited to capturing these nuanced experiences. Beyond contributing to theory, such insights can inform training programs that cultivate nurses’ capacity to reframe anxiety as a learning opportunity, enhance resilience in emotionally demanding communication, and support policy initiatives that promote advanced educational strategies for nursing practice.

The objective of this study is to elucidate the cognitive processes by which experienced oncology nurses regulate emotions when faced with anxiety and emotional conflict in communication with patients.

## Method

### Study Design

This study adopted a qualitative exploratory design to elucidate how experienced oncology nurses regulate their emotions in response to challenging clinical situations. Given the aim of gaining an in-depth understanding of the participants’ internal and often implicit experiences and cognitive processes, data were collected through semi-structured interviews.^21^^,^^22^ This approach enables participants to reflect on and narrate their experiences, which is essential for capturing the essential nature of lived experiences.

### Participants

Participants were oncology nurses holding nationally recognized specialty certifications in Japan, either as Cancer Nursing Clinical Nurse Specialists (CNS) or as Certified Nurses in Cancer Chemotherapy Nursing. The inclusion criteria stipulated a minimum of five years of clinical experience in oncology nursing to ensure that the participants could provide mature, practice-based reflections rather than accounts of novice-level anxieties.

Recruitment was conducted using snowball sampling. Initial participants were identified through professional oncology nursing networks, and subsequent participants were introduced via peer referrals. In each case, the researcher directly contacted referred nurses, provided study information, and obtained informed consent. A total of six participants were recruited between October and November 2019.

Prior to the interviews, participants received written information outlining the study purpose, data management procedures, and their right to withdraw at any time. Written informed consent was obtained from all participants. This study was approved by the Research Ethics Committee of the Japan Advanced Institute of Science and Technology.

### Data Collection

Interviews were conducted with a total of six nurses (Appendix A) between October and November 2019. All interviews were conducted in Japanese by the first author, following a semi-structured interview guide (Appendix B). To ensure the validity of the interview guide, a preliminary pilot was conducted with four nurses who had 5 to 6 years of clinical experience in oncology nursing. Based on their feedback, the clarity and appropriateness of the guide’s expressions and content were refined.^21^ These four nurses were not included in the main study. The average interview duration was 39.3 minutes. All interviews were audio-recorded and transcribed verbatim in Japanese.

### Data Analysis

Interview data were analyzed using the Steps for Coding and Theorization (SCAT), which involves (a) extracting notable words or phrases, (b) paraphrasing, (c) identifying underlying concepts, and (d) developing themes. Themes were subsequently woven into storylines, from which theoretical descriptions were derived.^23^^,^^24^ This method has been used in medical education and nursing research and is known for its clarity and rigor in qualitative analysis.^25^^,^^27^ These theoretical descriptions were then organized by grouping similar elements to identify shared characteristics among experienced oncology nurses. Data interpretation throughout the analysis was informed by metacognitive theory, specifically focusing on its two core components of “metacognitive knowledge” and “metacognitive regulation.”^17^^,^^18^

The adequacy of the sample was assessed through the concept of information power.^28^ Information power justified the sample size based on five parameters: (1) the study aim was narrow (focusing on emotion regulation processes); (2) participants were highly specific (certified oncology nurses with >5 years’ experience); (3) metacognitive theory guided the analysis;(4) interview dialogues were rich and contextually grounded; and (5)a cross-case analysis strategy was employed. Although cross-case analysis generally requires larger samples, the strong information power from the other parameters justified the adequacy of the present sample.

### Trustworthiness and Rigor

The first author (YI), a registered nurse with extensive oncology experience, conducted all interviews and analyses. While this professional background provided valuable interpretive insight into participants’ narratives, it also carried the potential risk of researcher bias. To address this, reflexive memos were maintained throughout the study to monitor the influence of the author’s assumptions and perspectives.^29^ All coding and categorization decisions were carefully documented and subjected to regular peer debriefing sessions with qualitative research experts. These sessions involved critical review of coding decisions, thematic structures, and interpretive consistency, thereby ensuring transparency and analytical rigor.^30^ All interview transcripts were produced in Japanese and translated verbatim into English by the first author for analysis. To preserve both linguistic accuracy and emotional nuances, the translated transcripts and representative quotations were critically reviewed and discussed in peer debriefing sessions with qualitative experts. This process helped ensure that the participants’ original meanings were faithfully conveyed in the reporting of the findings.

## Result

A total of 38 theoretical descriptions were generated through SCAT method. These were subsequently organized into two overarching themes: (1) anxiety and conflict in communication with cancer patients, and (2) metacognitive emotion-regulation strategies. The latter theme included two subthemes: (a) patient-oriented cognitive strategies”, and(b) self-oriented cognitive restructuring. Quotations are cited with participant number, gender, age, and years of oncology nursing experience.

### Anxiety and conflict in communication with cancer patients

Despite receiving specialized oncology nursing education and having more than five years of clinical experience, all participants described frequent experiences of anxiety and emotional difficulty in their interactions with cancer patients. The following are illustrative examples:

“I had a patient who needed to continue working to afford treatment, but the side effects would impair his sense of taste, making it difficult to work as a chef. I felt anxious about how to engage with him without diminishing his motivation for treatment.” [Participant 1, female, 30s, 16–20 years of experience]

“Undergoing stoma surgery would have allowed her to eat again, but as a young woman, she was concerned about the impact on her appearance. On the other hand, placing a central venous port would preserve her physical appearance but not fulfill her wish to eat. I struggled with how to face her treatment-related conflict without imposing my values.” [Participant 4, female, 50s, over 21 years of experience]

### Metacognitive emotion-regulation strategies

(a) Patient-oriented cognitive strategies

Participants engaged in cognitive-level interventions to respond to patients’ suffering. These included avoiding expressions that could evoke negative emotions and using language that supported the patients’ self-regulation of anxiety.

“When a patient complained, ‘I can't eat,’ I avoided saying ‘But you are eating,’ because I thought it might upset them. Instead, I chose to agree and empathize. Since the patient was concerned about weight loss, I tried to avoid reinforcing negative perceptions.” [Participant 3, female, 40s, over 21 years of experience]

“Patients often don’t know who to talk to about the struggles they experience during treatment. Even if they find it hard to speak directly to doctors, I tried to be someone they felt they could trust to connect them with the right support.” [Participant 4, female, 50s, over 21 years of experience]

(b) Self-oriented cognitive restructuring

Experienced nurses also described actively reinterpreting their own anxiety and inner conflict during difficult communication, using cognitive restructuring to regulate their responses.

“I find it difficult to deal with topics like treatment policy, where dilemmas can’t be fully resolved. Still, I try to be someone who can think things through together with the patient, so that they don’t feel alone.” [Participant 3, female, 40s, over 21 years of experience]

“It was initially very hard for me to face patients who expressed anger when treatment wasn’t going well. But just avoiding those situations won’t help me grow. I try to reinterpret the situation, telling myself that their anger must be grounded in some deeper reason.” [Participant 2, male, 40s, 16–20 years of experience]

## Discussion

This study qualitatively examined how experienced oncology nurses regulate their emotions in response to challenging clinical situations, and select responses in the face of difficult patient communication. The findings revealed that participants did not rely solely on reactive or situational coping; rather, they employed metacognition to recognize, interpret, and reconstruct their emotions. This enabled them to flexibly manage anxiety and conflict in interpersonal settings.

One illustrative case (Participant 2) was that of a nurse who reported initial difficulty in dealing with patients who expressed anger when treatment was not progressing as expected. However, he consciously reinterpreted the patient’s anger, telling himself that there must be a reason behind it. Through this reinterpretation, he broadened his capacity to respond, not by suppressing his own emotions, but by seeking a more structured understanding. Another nurse (Participant 3) reflected on dilemmas surrounding treatment decisions and described striving to be someone who could “think together with the patient” so that the patient would not feel alone. These instances illustrate a metacognitive redefinition of the nurse’s role through the reorganization of relational dynamics. Such processes align with the concepts of “monitoring” and “regulation” in the metacognitive frameworks.^17^^,^^18^ Collectively, these findings suggest that experienced nurses dynamically adjust their thoughts and emotions in accordance with situational demands, highlighting the potential of metacognitive skills as a form of practical expertise.^13^^,^^31^

### Comparison with previous studies

Previous qualitative studies on nurse emotion regulation have emphasized empathy, mindfulness, and communication skills training as strategies for managing stress and anxiety.^11^^,^^16^^,^^32^ While valuable for situational responsiveness, these approaches are predominantly response-focused, aiming to modulate emotions after they arise. Their effectiveness often remains limited to momentary relief and does not address the deeper cognitive reorganization required for enduring resilience.^14^

Research on emotional labor further highlights the risks of relying on surface acting and expressive suppression, which may help nurses maintain professional appearance but are consistently linked to emotional exhaustion and burnout.^9^^,^^33^^,^^34^ Such findings underscore that strategies focused on surface-level display without cognitive transformation are insufficient for sustainable practice.

Our findings extend these insights by showing that experienced oncology nurses engage in metacognitive reorganization—actively monitoring their own distress, reframing its meaning, and reorganizing their role in relation to patients. This goes beyond techniques such as cognitive reappraisal, which is often taught as a discrete strategy.^13^ Whereas reappraisal is typically framed as an intervention that can be practiced, the processes identified here reflect a spontaneous and embedded professional capacity that becomes integral to clinical expertise. This distinction highlights the novel contribution of our study: clarifying how metacognition underpins durable emotion regulation in real-world nursing practice.

### Theoretical contribution

The findings of this study enrich existing models of nurse emotion regulation by emphasizing the central role of metacognition in managing clinical anxiety. While previous models have largely prioritized response-focused strategies (e.g., mindfulness, empathic communication),^33^^,^^34^ our results demonstrate that antecedent-focused strategies—anticipating anxious reactions and preparing alternative interpretations—are equally critical for constructive engagement.

Situating these insights within Gross’s process model of emotion regulation clarifies that effective professional practice involves not only responding to emotions once they arise but also proactively shaping how they are appraised.^15^ This shift from response to anticipation underscores the necessity of expanding emotion regulation theory to explicitly incorporate metacognitive processes.^16^^,^^17^ In this way, the study provides a theoretical foundation for understanding emotion regulation as a metacognitive skill embedded in clinical expertise,^14^^,^^18^ suggesting that the ability to navigate complex, uncertain, and emotionally charged situations is a defining marker of advanced professional competence.

### Educational implications

The findings of this study highlight the potential for educational programs to incorporate structured reflection on nurses’ own anxiety as part of communication training.^12^ Conventional interventions such as empathy or mindfulness emphasize situational responsiveness, but they do not necessarily prepare nurses to reframe their own distressing experiences as opportunities for learning.^31^ An educational application might involve a module in which a patient reacts with anger to treatment uncertainty, and learners are guided to articulate their immediate emotional responses, examine the assumptions behind them, and consider alternative interpretations. This approach enables nurses to practice shifting from reactive coping to metacognitive reorganization of their thoughts.

Embedding such metacognitive reflection into ongoing professional education may help experienced nurses transform unavoidable emotional strain into constructive engagement with patients.^12^ This suggests that nursing education should not only aim to reduce emotional burden, but also to cultivate the ability to learn and grow through it.

### Transferability

Although this study focused on oncology nurses, the findings are relevant to other domains of healthcare where professionals face frequent emotional challenges, such as palliative care,^1^^,^^8^ psychiatry,^35^ pediatric care,^36^ and emergency medicine.^37^ In these contexts, clinicians are similarly required to manage patient suffering, uncertainty, and emotionally charged interactions, making the capacity for metacognitive reflection valuable across settings.

It is also important to recognize that certain contextual factors constrain transferability. The present findings were derived from experienced oncology nurses, who had both specialized knowledge and opportunities for long-term relationships with patients. The applicability of these processes is limited in settings where emotional intensity is lower, or where nurses have fewer chances to engage in sustained patient relationships. Furthermore, novice nurses require different forms of support, as their emotional regulation is often shaped by inexperience rather than by complex relational dilemmas.

### Limitations

The limitations of this study lie in both its scope and sample. The findings were derived from a small number of experienced oncology nurses with nationally recognized certifications in Japan.^28^ Their narratives reflect a highly specific clinical context characterized by intensive exposure to cancer-related emotional demands. As such, the strategies identified here are not representative of nurses in other specialties, less experienced practitioners, or those working in different healthcare systems.^30^

Although guided by metacognitive theory, the present findings cannot confirm whether similar processes operate in other professions. Future research should examine practitioners in roles that also involve high levels of emotional strain—such as palliative care, psychiatry, or emergency medicine—to explore how they recognize and reframe their own anxiety in challenging encounters. Such investigations would clarify whether the mechanisms identified here reflect a broader professional capacity for metacognitive regulation under emotional pressure.

## Conclusion

This study examined how experienced oncology nurses regulate their emotions in response to challenging clinical situations, particularly in patient communication. The findings demonstrate that experienced oncology nurses utilize metacognitive processes—rather than relying solely on reactive coping—to reinterpret anxiety and reorganize their professional role in challenging patient communication.^17^^,^^18^ These findings strongly suggest that fostering structured metacognitive reflection should be considered an essential element of educational programs in nursing to help practitioners cultivate durable coping strategies.^12–14,31^ For example, communication training could incorporate scenarios in which patients react with anger or uncertainty, guiding learners to articulate their immediate emotional responses, examine the assumptions underlying them, and practice reframing strategies. Such scenario-based reflection may help practitioners cultivate durable coping skills alongside empathy and mindfulness.

Future research should examine not only whether similar metacognitive processes are observed among healthcare professionals in other emotionally demanding contexts (e.g., palliative care, psychiatry, or emergency medicine) and at different levels of professional experience but also evaluate the effectiveness of educational interventions that explicitly integrate structured metacognitive reflection into training programs.

### Acknowledgments

The author would like to express sincere gratitude to Professor Mitsuru Ikeda of the Japan Advanced Institute of Science and Technology for his valuable advice on the development of the interview guide.

This study was supported by JSPS KAKENHI(Grant Number: JP23K19820).

### Conflict of Interest

The author declares that there is no conflict of interest.

## Supplementary material

Supplementary file 1 Appendix A. Detail of the participants and Appendix B. Interview Guide
