# Supplementary material for: Emotion regulation strategies of experienced oncology nurses: a qualitative study
Source: Int J Med Educ. 2025 Nov 26;16:181–7. doi: 10.5116/ijme.6921.a243 (PMC12700670; doi:10.5116/ijme.6921.a243)
Supplement: Supplementary file 1 — Appendix A. Detail of the participants and Appendix B. Interview Guide [file ijme-16-181-S1.pdf]

Appendix A

Detail of the participants

Participant	Age group	Sex	Years in oncology nursing
1	30s	Female	16-20
2	40s	Male	16-20
3	40s	Female	21-
4	50s	Female	21-
5	40s	Female	16-20
6	40s	Female	16-20

Appendix B

Interview Guide

1. Please describe a situation in which you experienced a challenge in communicating with a cancer patient.
2. What kind of difficulty or anxiety did you feel in that situation?
3. How did you cope with the anxiety you experienced?
4. Please explain the thought process that led you to adopt that coping strategy.
5. Has that experience increased your sense of anxiety when providing nursing care in similar situations?
6. What specific aspects of such situations make you feel anxious?
7. If you do not feel anxious in such situations, please describe any thoughts or strategies you use to prevent anxiety.
